# Proposing a New Method Based on Image Analysis to Estimate the Segregation Index of Lightweight Aggregate Concretes

**DOI:** 10.3390/ma12213642

**Published:** 2019-11-05

**Authors:** Afonso Miguel Solak, Antonio José Tenza-Abril, Francisco Baeza-Brotons, David Benavente

**Affiliations:** 1 Department of Civil Engineering, University of Alicante, 03080 Alicante, Spain; afonsosolak@gmail.com (A.M.S.); ajt.abril@ua.es (A.J.T.-A.); fbaeza.brotons@ua.es (F.B.-B.); 2 CYPE Ingenieros S.A., 03003 Alicante, Spain; 3 Department of Earth and Environmental Sciences, University of Alicante, 03080 Alicante, Spain

**Keywords:** lightweight aggregate concretes (LWAC), segregation, image analysis, ultrasonic tests, segregation index

## Abstract

This work presents five different methods for quantifying the segregation phenomenon in lightweight aggregate concretes (LWAC). The use of LWACs allows greater design flexibility and substantial cost savings, and has a positive impact on the energy consumption of a building. However, these materials are susceptible to aggregate segregation, which causes an irregular distribution of the lightweight aggregates in the mixture and may affect the concrete properties. To quantify this critical process, a new method based on image analysis is proposed and its results are compared to the well-established methods of density and ultrasonic pulse velocity measurement. The results show that the ultrasonic test method presents a lower accuracy than the other studied methods, although it is a nondestructive test, easy to perform, and does not need material characterization. The new methodology via image analysis has a strong correlation with the other methods, it considers information from the complete section of the samples, and it does not need the horizontal cut of the specimens or material characterization.

## 1. Introduction

Lightweight aggregate concrete (LWAC), a material widely used due to its many advantages, such as its low density, good thermal insulation, and fire resistance, has been extensively studied as both structural and non-structural material [1]. The use of LWACs allows greater design flexibility, considerable reductions of the dead loads, and substantial cost savings [2,3,4], and leads to improvements in the seismic resistance capacity of the structures [5]. As reported by Pla et al. [6], the use of LWAC does not establish significant differences in the fluid transport properties of lightweight concretes and when they are exposed to high temperature fluctuations, such as building fires, Young’s modulus of lightweight concretes decreases at a slower pace as the temperature increases than in conventional concretes [7]. Recently, as Energy Performance Construction Directives have been adopted by all European Union (EU) member states to promote the improved energy performance of buildings in the EU [8], structural LWAC, due to its good thermal properties, has presented itself as an alternative to conventional concretes since its use reduces the thermal bridging effects, as well as the building energy needs [9].

The replacement of part of the solid materials that make up LWACs with air, results in them having a lower specific weight when compared with normal-weight concretes. The most common materials used for this purpose are natural aggregates or artificial materials, with bulk densities below 300 kg/m^3^ [10]. However, during concrete vibration and transportation, LWAC may present aggregate segregation as a result of the density differences among its components. This phenomenon can be reduced or avoided, in order to adopt values of consistency and cohesion, as well as control the water–cement ratio; the proportion of fine aggregates; and the use of natural additions, such as silica fume [11,12]. 

According to Broomfield [13], to prevent the segregation phenomenon during concrete placing, the material should not be released from excessive heights or striking formwork systems, and must also be placed in uniform layers. Each layer must be completely vibrated before placing the next one, to reduce the amount of trapped air. The effort required for concrete compaction increases if the concrete’s consistency decreases [14]. As concluded by Solak et al. [15], the vibration time of concrete is a parameter that affects the segregation. The tendency for vertical movements of the lightweight aggregate (LWA) grows with the increase of the vibration energy applied to the material, and consequently, the concrete must have adequate cohesion to avoid segregation [16]. The segregation phenomenon causes numerous consequences, and it can affect both the mechanical and durability properties of the structures [17,18].

As reported by Panesar and Shindman [19], segregated concretes are more sensitive to the risk of cracking due to separation of the aggregates from the rest of the mixture, which entails surfaces rich in cement paste, areas that commonly suffer more from the contraction phenomenon. This effect can increase the vulnerability of reinforced concrete structures to the phenomenon of microcracking and reduce the resistance to the entry of moisture and ions. In the case of concretes exposed to frost–thaw cycles, it can lead to a high permeability and reduce the mechanical properties, affecting the integrity of the structure. A homogeneous distribution of the aggregates and a random orientation between them can improve the mechanical, impermeability, durability, and stability properties of concrete [20].

The publication of the Eurolightcon 1998 [21] highlights the importance of homogeneity between the components that constitute LWAC. Newman [22] pointed out that a good link between the mortar matrix and lightweight aggregates, and a similarity between the modules of the matrix and the aggregates, guarantee the efficiency of the matrix used. LWAC collapse does not occur due to displacement between the two phases, but as the result of collapse of the structure in the surroundings of the LWAs, which has a limited value of resistance. The fracture line crosses the aggregate grain, as in high-strength concrete, and the rupture occurs due to the fracture of the mortar matrix and the separation between the two phases, resulting in a line around the aggregates.

The aggregate volumetric fractions strongly affect the mechanical properties of LWAC, especially the compressive strength [23]. Usually, the mortar’s mechanical strength is substantially more elevated than that of the LWA, and a heterogeneous distribution of LWA in the mixture may strongly affect the concrete properties, which are frequently considered as homogenized values for design purposes [24]. An area presenting a high aggregate concentration may also lead to local pathologies when a long time period is considered [25]. The aspects mentioned above justify the experimental evaluation of segregation in concrete, adopting indexes for its quantification [26]. 

The most used and important methods to quantify the segregation phenomenon in concretes found in the literature will now be presented.

### 1.1. Method Proposed by Ke et al.

Ke et al. proposed a procedure for determining the segregation index of concretes (*SI_Ke_*) [27,28], subdividing the samples into four sections with the same height and adopting the densities obtained from the upper (*ρ_top_*) and lower (*ρ_botton_*) parts of a cylinder. When concrete segregates, the density in the upper section tends to reduce due to the vertical movement of LWAs toward the surface.

The segregation index is calculated according to Equation (1).

(1) $$SI_{Ke}=\frac{\rho_{top}}{\rho_{botton}}$$

If *SI_Ke_* = 1, the specimen is considered to be in a condition of uniformity. A value of less than 0.95 indicates that the concrete is at the start of segregation [28]. However, previous results indicate that this segregation index does not always reflect the real conditions of the sample, as well as the fact that it is sometimes difficult to find the areas of high concentrations of aggregates, which could demand the weighing and comparison of many specimens [29].

### 1.2. Method Proposed by Esmaeilkhanian et al. and Navarrete-Lopez

Adopting a particular case of the procedure proposed by Esmaeilkhanian et al. [30] and through an unbiased stereology technique based on count pointing [31,32], Navarrete-Lopez proposed a segregation index based on the volumetric fraction of aggregates at different heights of a sample [33]. 

Each sample was subdivided into three sections with the same height (top, middle, and bottom). For the top and bottom sections, the volume of LWA was calculated according to Equation (2):

(2) $$V_{ai}=\frac{P_{ai}}{P_{refi}}\times 100\%,$$

where *P_ai_* is the sum of the points intersecting the LWA in section *i*, *P_refi_* is the sum of the points intersecting section *i*, and *V_ai_* is the LWA volume fraction of section *i*.

To estimate the segregation, the volumetric index (*VI*), proposed by Esmaeilkhanian et al. [30], was calculated according to Equation (3):

(3) $$VI\left(\%\right)=2\times \frac{\left|V_{at}-V_{ab}\right|}{V_{at}+V_{AB}}\times 100\%,$$

where *V_at_* and *V_ab_* are the LWA volume fraction of the top and bottom sections, respectively.

The studies of Kwasny et al. [31] suggested that LWAC may be considered as non-segregated when the VI is lower than 20%. Esmaeilkhanian et al. [34] studied the dynamic segregation of self-compacting concrete and proposed the value of *VI* = 25% as the limit for segregation. Navarrete-Lopez [11] proposed a range of segregation levels classified into five degrees (Table 1).

### 1.3. Method Proposed by Solak et al.

Unlike most conventional methods and tests, ultrasonic pulse velocity methods do not significantly affect the microstructure when they are used to evaluate concrete characteristics. The methods that use the propagation of waves and their interaction with concrete are among the most used and important nondestructive methods for the study of concrete [35,36]. Besides, several studies have found correlations between the density of different materials and the speed of propagation of ultrasonic pulses in their interior. Chen et al. [37] found empirical correlations between P-wave velocities (VP) and the basalt density and porosity. Their results show a linear relationship between the P-wave velocity and the dry density of the samples, with a coefficient of determination of R = 0.9078.

Benaicha et al. [38] evaluated the segregation of self-compacting concretes, adapting a technique based on ultrasonic velocities. To analyze the homogeneity and quality of the concretes, ultrasonic velocities were measured at several points of a column of concrete in a semi-fresh state. They pointed out that the methods of ultrasonic measurement applied to studies of concretes are complicated because they depend on many variables, including the porosity, heterogeneity of the types of cement, aggregates, and additives, whose particles have dimensions that vary from nanometers to centimeters. Even with the complexity of data interpretation, Benaicha et al. [38] affirmed that the results obtained by ultrasound and empirical methods were similar, and concluded that, in the laboratory, ultrasound methods could be used instead of empirical methods to evaluate the static stability of self-compacting concretes.

In previous work, Solak et al. [39,40] proposed a segregation index based on ultrasonic pulse velocity (UPV) measurements (*SI_UPV_*). *SI_UPV_* calculates the segregation considering the UPVs measured in the upper (*UPV_top_*) and lower (*UPV_bottom_*) slices of the specimens. The results of these works [39,40], based on the clear relation between UPVs and concrete densities, indicated that UPV measurements are an easy and non-destructive way to evaluate the concrete segregation in hardened samples, once the reduction of the density of the upper sections caused by vertical movement towards the surface of the LWA leads to a reduction of the UPVs. The index is calculated according to Equation (4) and *SI_UPV_* = 1 is considered perfect uniformity.

(4) $$SI_{UPV}=\frac{UPV_{top}}{UPV_{bottom}}$$

### 1.4. The Use of 2D Images to Represent 3D Phenomenon

Techniques based on image processing have been previously used to evaluate LWAC sections, by analyzing the particle size distribution of aggregates [41], and have also been applied to the analysis of segregation in LWAC [29]. Both cases adopt strategies based on the assumption that the amount of aggregates identified by image analysis on a concrete section tends to be correlated to the respective aggregate’s volumetric fraction in the mixture [29]. Mouton [42] demonstrated that the area of an object on arbitrary surfaces cut through the reference space is proportional to the 3D volume of the object in the reference space.

### 1.5. The Aim of the Study

The main objectives of this study are to propose a new index that represents unidirectional segregation in concrete samples and evaluate the data from the complete section of a sample by adopting image analysis technics. To validate this methodology and estimate the segregation index, four other methods using standard density measurements, ultrasonic velocity measurements, and other image analysis technics were used.

## 2. Materials 

During the experimental campaign, LWACs with target densities of 1700 and 1900 kg/m^3^ were produced following the Fanjul method [43]. The Fanjul [43] method was designed for dosing lightweight and heavyweight concrete by fixing the density before concrete production. According to this method, the concrete aggregates can be obtained in five steps, as follows: Step (a) obtain the absolute aggregate volume and calculate the reference concrete; step (b) determine the initial n-2 aggregate volume; step (c) calculate the actual n-2 aggregate volume; step (d) determine the masses and volumes of the two aggregates with the lowest density; and step (e) obtain all the volumes of the concrete constituents. According to this method, a target concrete density can be established and a one-meter cubic is exactly filled, irrespective of the number of aggregates used and their density, with a high precision.

Eight different concrete mixtures were produced considering different types of LWA, different types of vibration (one or two layers), and different theoretical densities. Table 2 includes the concrete mix proportions, and Table 3 shows their manufacturing characteristics.

CEM I 52.5 R cement with a real density of 3176 kg/m^3^ was achieved for all the mixtures and four types of expanded clay were adopted as lightweight aggregates. Their physical properties are detailed in Table 4, and their size distributions are detailed in Table 5. The bulk density of the LWAs was obtained following the procedure detailed in the standard UNE EN 1097-3 [44].

The density of the particles in the dry state was also determined by the methodology proposed by Fernández-Fanjul et al. [45] and the absorption of water at 24 h, according to the UNE EN 1097-6 [46] (pre-dried particles and in distilled water). The absolute density of the aggregates was determined by a helium pycnometer and the granulometric fractions of the aggregates according to the UNE EN 933-1 [47].

Before mixing, to avoid the loss of water from kneading by absorption, the LWAs were water-presaturated. Following the recommendations of Fanjul et al. [48] and aiming to maintain a constant effective a/c ratio of 0.6, the water content and surface water content of the LWA were determined and corrected during the mixing. Characterization of the density, porosity, and water absorption of the mortar was obtained for each concrete prismatic mortar sample of 40 × 40 × 160 mm, according to the UNE EN 196-1 [49]. Curing of the specimens was conducted in the water at a temperature of 20 ± 1 °C, and their values were determined at 28 days of age (Table 6).

## 3. Experimental Methodology 

**Figure 1:**
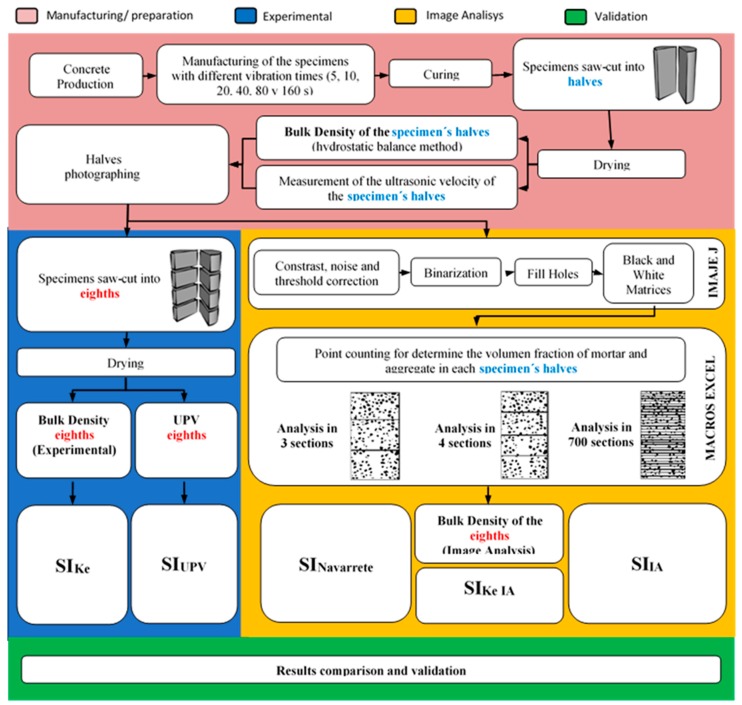
Experimental methodology: manufacturing and preparation of the concrete specimens (red color); experimental phase (blue color); image analysis phase (yellow color); and comparison and validation (green color).

The methodology is represented in the diagram of Figure 1. This methodology was divided into four main sections: (i) manufacturing of the concrete specimens (red color); (ii) experimental phase (blue color); (iii) image analysis phase (yellow color); and (iv) comparison and validation (green color).

### 3.1. Manufacturing of the Concrete Specimens

The concrete was manufactured by considering the following variables when making the specimens (cylinder of a 150 mm diameter and 300 mm height). The compaction was performed using an electric needle vibrator of 18,000 rpm/min and a needle diameter of 25 mm. The specimens were vibrated with six different times (5, 10, 20, 40, 80, and 160 s) in one or two layers (Figure 1).

**Figure 2:**
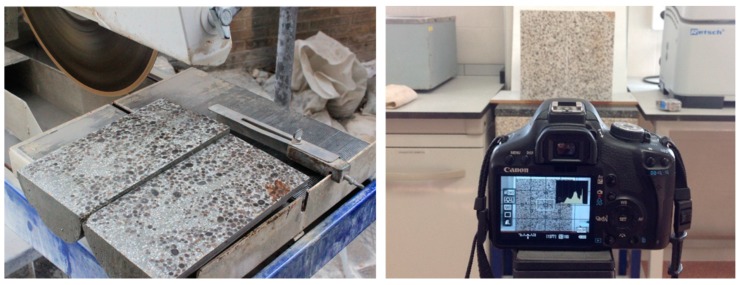
Left: saw cutting of the specimens into halves. Right: photographing of the halves.

Samples were cured in the water at a temperature of 20 ± 1 °C for 28 days. The samples were saw-cut through their longitudinal axis (Figure 2, left), their bulk densities were determined by the hydrostatic balance method, and their P ultrasonic velocities were measured. Subsequently, their sections were photographed (Figure 2, right) for image analysis.

The photographs were taken in a natural light environment using a Canon EOS 500D camera, with a resolution of 4752 × 3168 pixels, ISO-100, an aperture of f/5.6, and an exposure time of 1/3 s, without a flash. The two halves of each cylinder were photographed at the same time.

### 3.2. Experimental Phase. Density and Ultrasonic Pulse Velocity

Each specimen’s halves were saw-cut into four equal subsections, resulting in eighths, and their bulk densities and compressive wave velocities were determined. Using the density values of the upper and lower subsections, the segregation index was obtained according to the methodology indicated by Ke (*SI_Ke_*) [28].

Considering the existence of a relationship between the UPVs and the densities of the material, using the UVPs of the upper (UPV_top_) and lower (UPV_bottom_) subsections, a second experimental segregation index was estimated according to the methodology presented by Solak et al. [39] and calculated according to Equation (4), previously described (in Section 1.3). The compressional wave velocity or ultrasonic pulse velocity (UPV) was obtained using the direct transmission configuration employing Panametric transducers (54 kHz).

### 3.3. Image Analysis Phase

The images of the sections (halves) were used to calculate the segregation index according to Ke [28], this time using the image analysis technique (Appendix A). To process the images and determine the black and white matrices (binarization), ImageJ, a freeware software platform, was used. The density and segregation index were calculated using the point-counting method. The treatment of images and the determination of the matrices were performed according to the following procedure.

#### 3.3.1. Initial Treatment of the Images

**Figure 3:**
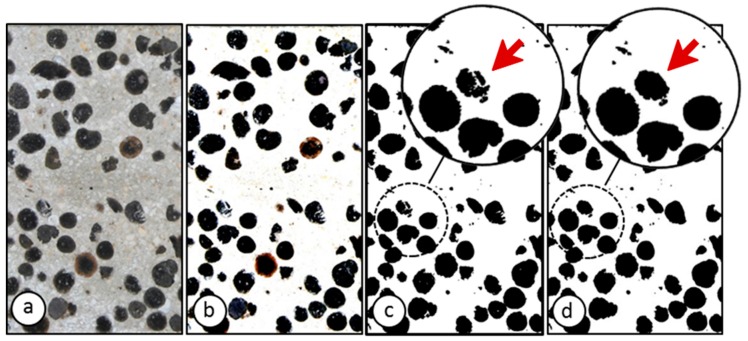
Initial treatment of images and binarization conducted in imageJ: (**a**) Original image; (**b**) contrast, threshold, and noise adjustments; (**c**) binarization; (**d**) fill holes.

The same treatment was performed for all the specimens. First, from the original image, the perspective was corrected, with the aim of eliminating any errors caused by inclinations of the camera angle or the surface where the specimens were located. Once the perspective correction had been completed, the contrast and threshold were adjusted, the noise was reduced (Figure 3b), the image was binarized (Figure 3c), and the internal voids of the aggregates were filled using ImageJ (Figure 3d).

#### 3.3.2. Binarizatio

**Figure 4:**
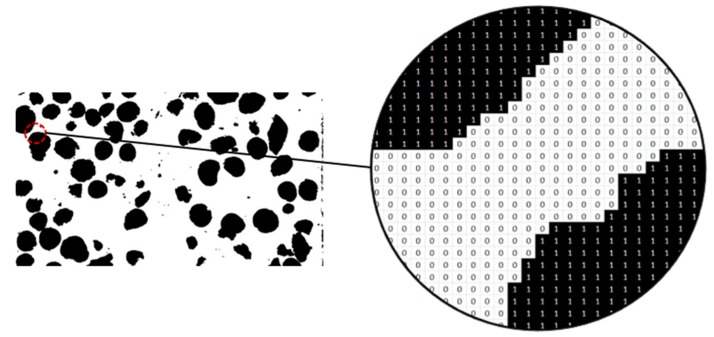
Black and white matrix.

The binarization of images distinguishes between LWA and mortar. This binary code relates the black color, with a numerical value equal to 1, to LWA, whereas the white color, with a numerical value equal to 0, is equivalent to parts of the mortar matrix (Figure 4).

#### 3.3.3. Data Processing of Black and White Matrices 

**Determination of the volumetric fraction of aggregates and mortar, via image analysis:** The volume fraction or the percentage of aggregates (or mortar) was estimated by counting of the number of black (aggregates) elements and number of white elements (mortar) in a particular area of the matrix. This count was done by adopting Macros using Visual Basic in Microsoft Excel^®^.

**Determination of the density, via image analysis:** The percentage of each material in each section was quantified as previously described. Since the densities of the mortar matrix and the LWAs were known, the density of the section was determined ($\rho_{secction}$) and analyzed by means of Equation (5), where *N_mortar_* is the percentage of mortar pixels present in the analyzed area, *N_LWA_* is the percentage of LWA pixels present in the analyzed area, ρ_mortar_ is the bulk density of mortar at 28 days of age (Table 6), and *ρ_LWA_* is the dry density of the LWAs (Table 4).

(5) $$\rho_{secction}=\frac{N_{mortar}\times \rho_{mortar}+N_{LWA}\times \rho_{LWA}}{N_{mortar}+N_{LWA}}$$

This procedure employed to determine the densities of the specimens by image analysis has been used in other publications of Solak et al. [16,50,51].

**Segregation index proposed by Ke adapted for obtaining data via Image Analysis (IS_Ke AI_):** The black and white matrices, related to the specimen halves, were horizontally separated into four equal-sized subsections, equivalent to the specimen eighths. The upper and lower subsections were analyzed separately, and the densities of these subsections were obtained via image analysis. From the densities obtained for the eighths, the segregation indexes were calculated using the method proposed by Ke [28] via image analysis. The method was applied to 101 specimens, equivalent to 202 black and white matrices and 808 specimen eighths, of which 404 (upper and lower) were used to calculate the segregation index according to Ke [28].

**Segregation index proposed by Navarrete-Lopez (SI_Navarrete_), obtained via Image Analysis:** Using the same matrices, other segregation indexes were calculated. To obtain the segregation indexes using the method proposed by Navarrete-Lopez [33], the black and white matrices (halves) were horizontally separated into three subsections of the same size, representing specimen sixths. For the top and bottom subsections, the volume fraction of LWA was estimated using the point-counting technique. For a randomly positioned point grid, with points disposed every 0.57 mm, the elements of each color found in each of the three sections was counted to obtain the volumetric fraction of the specimen sixths, and subsequently, the segregation indexes using the method proposed by Navarrete et al. [33] were estimated. The method was applied to 101 specimens, equivalent to 202 black and white matrices and 606 specimen sixths, of which 404 (upper and lower) were used to calculate the segregation index according to Navarrete et al. [33].

## 4. Segregation Index Proposed in this Study (SI_IA_), Obtained via Image Analysis

One of the objectives of this work is the proposal of a new segregation index, obtained through image analysis, which does not require previous characterization of the materials and which evaluates the phenomenon in a more precise way, considering the data of 100% of the cross-section of the specimen analyzed. To obtain the segregation index according to Solak (*SI_IA_*), the following methodology must be applied.

### 4.1. Calculation of the Global Aggregate Index (GAI), Calculated for the Whole Surface Analyzed

The GAI represents the volumetric fraction of aggregates presented in a complete cross-section of a specimen and is calculated according to Equation (6):

(6) $$GAI=\frac{N_{LWA}}{N_{LWA}+N_{M}},$$

where $N_{LWA}$ represents the total elements (pixels) classified as aggregates, found in a complete cross-section, and $N_{M}$ represents total elements (pixels) classified as mortar, found in a complete section.

**Figure 5:**
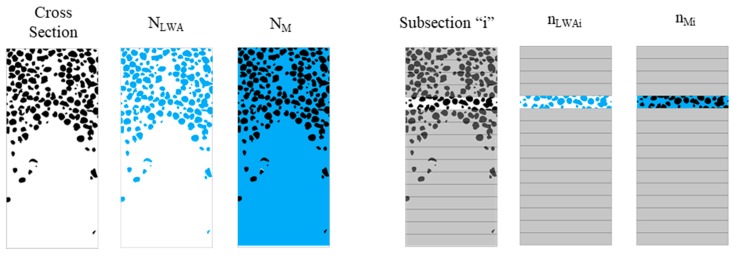
Cross-section (**left**) and subsection (**right**) of a specimen.

The section of the specimen was subdivided into “i” subsections (Figure 5) that were analyzed separately, analogous to Ke’s [28] (four subsections) and Navarrete et al.’s [33] (three subsections) methods. 

### 4.2. Local Aggregate Index (LAI), Calculated for Each Subsection

In each subsection, we applied the same procedure applied to calculate the GAI, although locally. The LAI—Equation (7)—represents the volumetric fraction of aggregates present in a certain subsection that belongs to a cross-section of a sample:

(7) $$LAI_{i}=\frac{n_{LWA_{i}}}{n_{LWA_{i}}+n_{M_{i}}},$$

where $n_{LWA_{i}}$ represents the total elements (pixels) classified as aggregates, found in a subsection “i”, and $n_{M_{i}}$ indicates the total elements (pixels) classified as mortar, found in subsection “i”.

If the LWAC does not present segregation, there is a homogeneous distribution of aggregates in the sample, and consequently, the *LAI* values of the “*i*” subsections should be equal to the *GAI*.

### 4.3. Local Absolute Difference (LAD), Calculated for Each Subsection

The absolute difference between the LAI of each subsection and the GAI—Equation (8)—quantifies how far that subsection is from the ideal situation of homogeneity. In other words, when the LAD of a subsection has greater values, higher segregation occurs in this subsection.

(8) $$LAD_{i}=\left|LAI_{i}-GAI\right|$$

Analyzing the local difference (LD), without considering that the values are absolute, positive results indicate that in the subsection analyzed, there is an excess of aggregates and negative results indicate that there is an excess of mortar.

### 4.4. Local Distribution Coefficient (LDC) = Average of the Local Absolute Differences

The local distribution coefficient (*LDC*) is the average of the “i” LDAs calculated for the “i” subsections analyzed. As 100% of the cross-section of the concrete specimen is analyzed, there will always be an aggregate compensation between the subsections. That is, the aggregates that move out of a certain sub-section will always be relocated to another subsection. Therefore, if we do not use the absolute value for the calculation of the LDAs (using LDs), the *LDC* will always be null. The *LDC* is calculated with Equation (9):

(9) $$LDC=\frac{\sum_{1}^{i}LDA_{i}}{i},$$

where i is the total number of subsections analyzed.

### 4.5. Segregation Index Obtained via Image Analysis (SI_IA_)

The LDC is an indicator of segregation, but its values are very susceptible to variations in the GAI. For example, two situations with different GAIs, but with similar degrees of segregation, can present an important dispersion among their LDCs.

**Figure 6:**
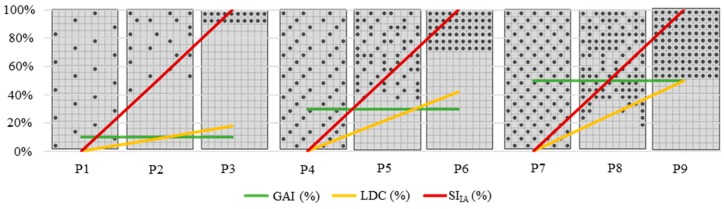
Synthetic specimens. P1, P2, and P3: Global aggregate index (GAI) = 10%; P4, P5, and P6: GAI = 25%; P7, P8, and P9: GAI = 50%.

To illustrate this statement, synthetic specimens with different GAIs were developed (Figure 6). More detailed figures can be seen in the supplementary material (from Figure S1 to Figure S4). We established a situation where the homogeneous distribution of aggregates that occurs is equivalent to a zero-segregation index, *SI_IA_* = 0% (P1, P4, and P7). The situation where the maximum displacement of aggregates occurs is when 100% of the aggregates are concentrated at the top of the specimen. This situation was considered as the maximum segregation hypothesis, *SI_IA_* = 100% (P3, P6, and P9).

In total, 45 synthetic specimens with different GAIs and different geometric proportions were simulated with the intention of seeking a pattern that would provide a correction coefficient valid for any specimen, with any *GAI*, and a segregation scale whose minimum value was 0% and maximum value was 100%. For all of them, the correction coefficient (*K*) evaluated all the hypotheses within the same scale and can be defined as Equation (10).

(10) $$K=\frac{1}{2\times GAI\times \left(1-GAI\right)}$$

Therefore, the segregation index (*SI_IA_*) could be determined by the Equation (11) or by Equation (12).

(11) $$SI_{IA}=K\times LDC$$

(12) $$SI_{IA}=\frac{LDC}{2\times GAI\times \left(1-GAI\right)}$$

Microsoft Excel^®^ was used to process the data of the black and white matrices. Each section generated a data matrix organized into 701 rows and 326 columns, equivalent to 700 × 325 pixels for each of the photographed images (reduced in size if compared to the original images). To facilitate the processing of data and obtain more secure results, macros were developed using Visual Basic [50]. Figure S1 shows the minimum values of subsections needed for a good accuracy.

## 5. Results and Discussion

The same procedure used by Solak et al. [50] to verify the possibility of using image analysis to evaluate the segregation of LWACs was carried out in this study, but considering an even wider range of data (all the data are presented in Table S1 in the supplementary material). Two validation criteria were applied to evaluate the effectiveness of the image analysis methods.

### 5.1. Validation Criteria 1—Density: Experimental Procedure vs. Image Analysis Methodology

**Figure 7:**
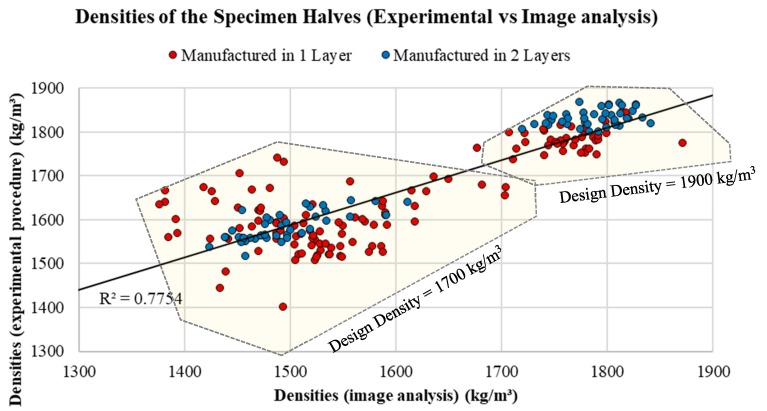
Densities of the specimen “halves”: data obtained experimentally versus data obtained by image analysis.

**Figure 8:**
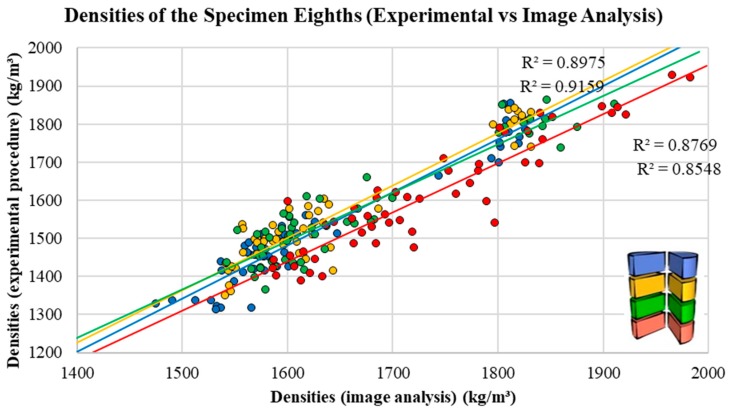
Densities of the specimen “eighths”: data obtained experimentally versus data obtained by image analysis.

As shown in Figure 7 (specimen halves) and Figure 8 (specimen eighths), the density values obtained with image analysis techniques are very close to the density values obtained experimentally. The method was shown to be a viable alternative for both the analysis of specimen halves (208 samples), with R^2^ = 0.754, and the analysis of specimen eighths (832 samples), with R^2^= 0.7585. The results demonstrate that this technique may be suitable for estimating the density of LWACs, if the density values of both the mortar and the LWA are known.

### 5.2. Validation Criteria 2—Segregation Index According to Ke: Experimental Procedure vs. Image Analysis

**Figure 9:**
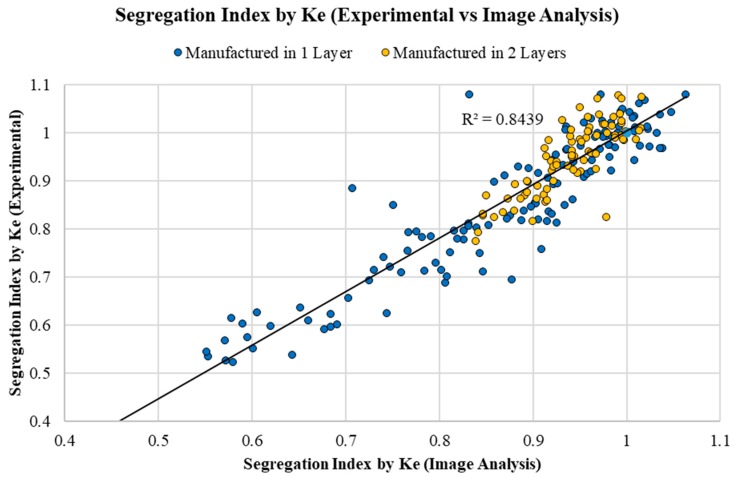
Segregation index proposed by Ke [28]: data obtained experimentally versus data calculated by image analysis.

As shown in Figure 9, the experimental values of the *SI_Ke_* [28] are very close to those obtained using the image analysis technique for 208 samples and with R^2^ = 0.8439. As a conclusion, this technique may be adequate for quantifying the phenomenon of the segregation of LWACs using the values of density of the mortar and LWA to estimate a segregation index based on the methodology proposed by Ke [28].

### 5.3. Comparing the Proposed Segregation Indexes with Segregation Indexes Proposed by Other Authors

**Figure 10:**
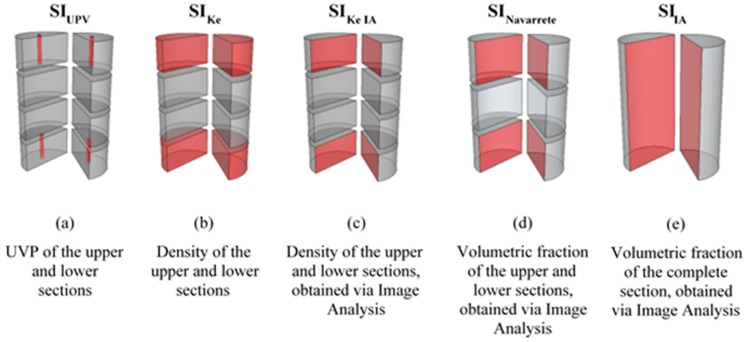
Origin of the data used for calculating each segregation index.

**Figure 11:**
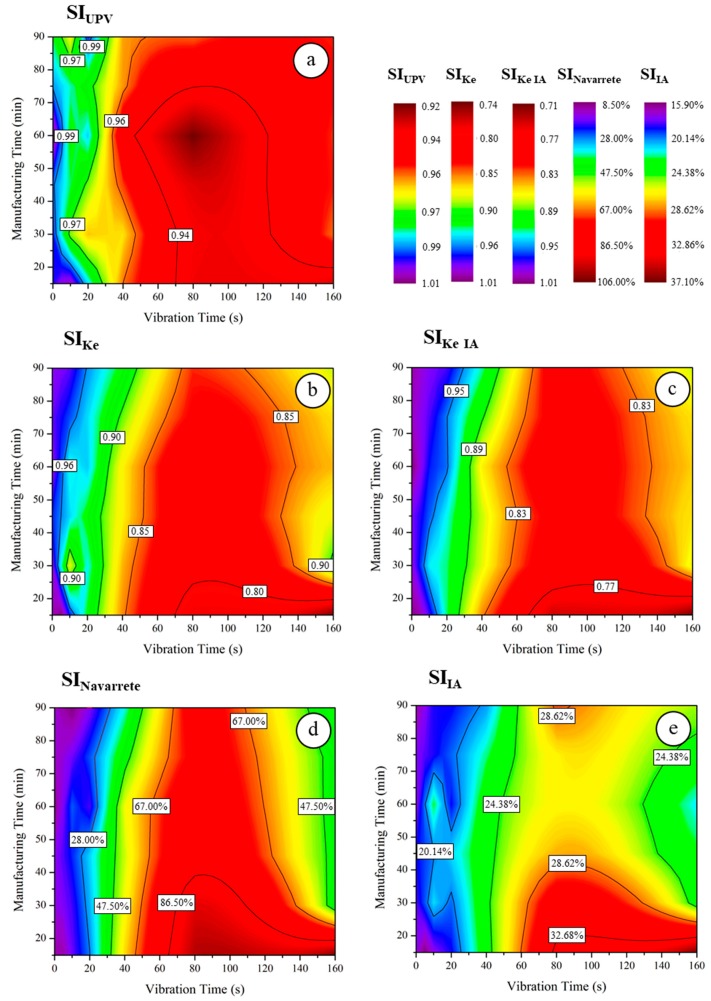
Results for SI_UPV_ (**a**), SI_KE_ (**b**), SI_KE IA_ (**c**), SI_Navarrete_ (**d**), and SI_IA_ (**e**), according to the manufacturing and vibration times.

The origin of the data used for calculating each segregation index are summarized in Figure 10. The results referring to the segregation indexes obtained for each vibration time and manufacturing time are represented in Figure 11 and collected in Table S1 (supplementary material).

Segregation was quantified using different methods, both by experimental procedures and by image analysis. One of the objectives of the study was to verify the feasibility of applying these methods, and the correlations between their results. For this propose, we performed a statistical study using Pearson correlation coefficients, r, comparing the results of the different indexes. A total of 208 observations were made, referring to the 104 samples studied (divided into two sections). The minimum, maximum, average values, and standard deviation of the data studied are presented in Table 7.

The segregation index calculated using the ultrasonic pulse velocity data presented lower correlations with all other segregation indexes and can be classified as having a “moderate” or “strong” correlation using the Evans Scale [52], directly proportional to SI_Ke_ and SI_Ke IA_ and inversely proportional to SI_Navarrete_ and SI_IA_. Although UPV presented a lower accuracy than the other studied methods and did not analyze the data of the central zone of the samples, it presented the advantages of easy data collection and the lack of a need to determine the density of materials (LWA and mortar).

As seen in the Pearson correlation matrix (Table 8) and according to the Evans classification [52], there is a “very strong” correlation (r = 0.919) between SI_Ke_ and SI_Ke IA_. These results are consistent with what has been presented in Section 5.1 and Section 5.2.

The same situation can be observed for the correlations SI_Ke_–SI_IA_ and SI_Ke IA_–SI_IA_. According to the Evans scale [52], the coefficients of r = −0.865 and −0.822, for the experimental method and the image analysis method, respectively, indicate a “very strong” and inverse correlation between the two indexes. SI_Ke_ and SI_Ke IA_ present the same behavior when their results are compared with the vibration time applied: their values decrease as the vibration time increases. SI_UVP_ has values slightly higher than SI_Ke_ and SI_Ke IA_. The difference is more pronounced in concretes vibrated in one layer, mainly in those that have been subjected to high vibration times.

The method proposed by Ke et al. [27,28] presents good correlations with the other methods and does not require the “vertical and horizontal cut” of the specimens. However, it does require previous characterization of the materials (determination of the dry density of the upper and lower sections of the samples), and its accuracy depends on a good distinction between the aggregates and the mortar matrix (image).

The accuracy of the methods conducted by image analysis depends on a series of factors related to the way in which the data is captured and treated. During the data collection with respect to photographing the sections, good-quality images, ambient light, and most importantly, a good distinction between the mortar matrix and the coarse aggregates, are parameters that must be taken into consideration. During the stages of image processing, shadows, voids, and noise make it difficult to classify each pixel as “aggregate” or “mortar”. At this point, a small part of the data will inevitably be lost, and that is reflected as the difference between the method proposed by Ke et al. [27,28] and the method proposed by Ke, performed via image analysis.

SI_Ke_ and SI_Ke IA_ presented similar values, with small dispersions in lower vibration times (under 40 s). These similitudes can be identified by comparing Figure 10b,c. When there is great homogeneity inside the specimen, the upper zone and lower zone are similar and are quantified with the same experimental conditions and image treatment. As the segregation index represents the relationship between the values obtained from the two sections, with low vibration times, the errors do not significantly affect the final results, although, as the vibration time increases, the difference between both methods becomes more pronounced. With the displacement of the aggregates to the upper zone, the treatment of images is more affected in this area, making it more difficult to identify what is a mortar matrix and what is LWA. In the lower zone, the situation is the opposite: the mortar matrix is predominant, and the classification of each pixel becomes easier.

The correlations *SI_Ke_*–*SI_Navarrete-Lopez_* and *SI_Ke IA_*–*SI_Navarrete-Lopez_* are considered “very strong”, according to the Evans scale [52]: r = −0.907 and −0.917, respectively. In this case, the Pearson coefficient indicates that the correlation is inversely proportional, which means that, the greater the segregation index of Ke, the lower the segregation index of Navarrete-Lopez.

The method proposed by Navarrete eliminates the step of the “horizontal cut” of the specimens and the need for material characterization. This method still does not analyze the data of the central zone of the sample, and its accuracy also depends on a good distinction between the aggregates and the mortar matrix.

*SI_Navarrete_* and *SI_IA_* have shown the best correlation between the studied indexes (r = 0.925), which is “very strong” according to the Evans scale [52]. The results of both methods are presented in the same magnitude, and their data come from the same source: photographs of the cross-sections of the samples. Both the *SI_Navarrete-Lopez_* and *SI_IA_* results show similar behaviors: their values increase as the vibration time increases. As their scales are expressed as a percentage and are inverse to the scales of three other methods, an increasing correlation is expected. At first, the values of the results of the Navarrete-López method seem to be higher than the results obtained with SI_IA_, but it is important to bear in mind that, although both are expressed as percentages, they are represented by different scales: the SI_Navarrete_ varies from 0% to 200%, and the SI_IA_ varies from 0% to 100%.

The new method conducted via image analysis proposed in this paper has a strong correlation with the other methods, considers information from the complete section of the samples, does not need the “horizontal cut” of the specimens, and does not request material characterization. Its main disadvantage is that its accuracy also depends on a good distinction between the aggregates and the mortar matrix. Table 9 compares the different methods used in the research.

## 6. Conclusions

This study presents an experimental investigation on segregation in lightweight aggregate concretes (LWAC), comparing different methods to estimate the segregation phenomenon of LWAC samples. From the results presented in this study, the following conclusions can be drawn:

- The calculation of densities and segregation indexes with the proposed image analysis method has been shown to be a reliable alternative to the experimental method, since the results obtained with the two methods show little dispersion among themselves;
- In the laboratory procedures, the methods of image analysis were shown as an efficient option for quantifying the proportion of materials of the specimens. During the procedure of image analysis, drying and weighing stages are not necessary, which results in a saving of time in the research;
- With the image analysis method, it becomes possible to section the specimen into a greater number of zones and thus determine a segregation index that is not limited to only the eighths of the specimen. A greater number of sections leads to obtaining a segregation index that is more realistic;
- The new method for the quantification of segregation proposed in this work (*SI_IA_*) was shown to be an effective option for the quantification of the phenomenon. In addition, it was shown to be a viable option for cases in which segregation does not occur at the top/bottom of the specimens, once it considers all the data of the section;
- The accuracy of the methods conducted by image analysis depends on a series of factors related to the way in which the data is captured and treated. During the photographing of the sections, good-quality images, the lighting conditions, and a good distinction between the mortar matrix and the coarse aggregates are parameters that must be taken into consideration. During the treatment of images, shadows, voids, and noise make it difficult to classify each pixel as “aggregate” or “mortar”. At this point, a small part of the data inevitably ends up being lost, and that is reflected as the difference between *SI_Ke_* and *SI_Ke AI_*;
- The comparative study carried out with the UPV shows values consistent with the data obtained by image analysis. The increase in densities of the lower eighths due to the phenomenon of segregation caused an increase in speeds. This method presented the lowest correlations when compared with the other methods, although it has been shown to be the fastest method for determining the segregation index.

## Figures and Tables

**Table 1:** The volumetric index (*VI*) range of segregation levels proposed by Navarrete-Lopez [11].

Segregation Level	*VI* Range (%)
None to slight	0–40
Moderate	40–80
Severe	80–120
Slightly stratified	120–160
Highly stratified	160–200

**Table 2:** Mix proportions used to produce 1 m^3^ of concrete.

Concrete	Cement (kg/m^3^)	Water (kg/m^3^)	Fine Coarse (kg/m^3^)	LWA (kg/m^3^)
LWAC1	350	210	723.9	416.2
LWAC2	350	210	1046.0	294.0
LWAC3	350	210	991.1	148.9
LWAC4	350	210	1234.8	105.2
LWAC5	350	210	991.1	148.9
LWAC6	350	210	938.6	201.4
LWAC7	350	210	723.9	416.2
LWAC8	350	210	662.0	473.0

**Table 3:** Manufacturing characteristics of the studied concretes.

Concrete	Samples (ud)	Theoretical Densities (kg/m^3^)	Vibration	Type of LWA
LWAC1	20	1700	two layers	Arlita Leca HS
LWAC2	20	1900	two layers	Arlita Leca HS
LWAC3	20	1700	one layer	Arlita Leca M
LWAC4	20	1900	one layer	Arlita Leca M
LWAC5	6	1700	one layer	Arlita Leca M
LWAC6	6	1700	one layer	Laterlite LTM
LWAC 7	6	1700	one layer	Arlita Leca HS
LWAC 8	6	1700	one layer	Laterlite LTHS

**Table 4:** Characteristics of aggregates and the methods/standards used for testing.

Property	Method	Arlita Leca M	Laterlite LTM	Arlita Leca HS	Laterlite LTHS	Fine Coarse
Dry particle density (kg/m^3^)	Acord [45]	482	613	1019	1118	2688
Bulk density (kg/m^3^)	UNE EN 1097-3 [44]	269	276	610	676	1610
24 h Water absorption (%)	UNE EN 1097-6 [46]	36.6	29.55	12.2	11.05	0.12
Granulometric fraction (d_i_/D_i_)	UNE EN 933-1 [47]	16/6	12/4	12/4	12/4	0/4

**Table 5:** Aggregates’ grain size distribution.

Size (mm)	Sieving Fraction (%)				
	Arlita Leca M	Laterlite LTM	Arlita Leca HS	Laterlite LTHS	Fine Coarse
16	99.91	100.00	100.00	100.00	100.00
12	95.00	99.96	98.36	95.05	100.00
8	5.87	76.50	68.71	65.33	100.00
6	3.21	45.11	41.43	37.81	100.00
4	2.97	4.46	5.12	13.56	99.86
2	2.93	1.04	0.91	2.22	72.27
1	2.91	1.04	0.76	0.58	47.18
0.500	2.88	1.04	0.75	0.47	32.32
0.250	2.80	1.04	0.74	0.43	23.15
0.125	2.52	1.03	0.70	0.40	17.24
0.063	1.99	0.98	0.65	0.38	14.00

**Table 6:** Mortar characterization for each concrete.

Mortar	Age (days)	Density (kg/m^3^)	Absorption (%)	Porosity (%)
M1	28	2022	12.16	24.63
M2	28	2104	10.01	21.11
M3	28	2061	10.78	22.25
M4	28	2104	10.01	21.11
M5	28	2061	10.78	22.25
M6	28	2050	11.03	22.65
M7	28	1979	12.93	25.63
M8	28	1955	13.57	26.57

**Table 7:** Characteristics of the data used in the statistical study.

Variable	Observations	Minimum	Maximum	Average	Standard Deviation
SIUVP	208	0.820	1.106	0.967	0.053
SIKe	208	0.552	1.063	0.898	0.114
SIKe AI	208	0.522	1.115	0.890	0.138
SI_Navarrete-Lopez_ (%)	208	0	199	46	50
SIIA (%)	208	11	65	23	10

**Table 8:** Matrix of Pearson correlations between the segregation indexes studied.

Variables	SI_UPV_	SI_Ke_	SI_Ke IA_	SI_Navarrete-Lopez_	SI_IA_
SI_UVP_	1	0.638	0.572	−0.596	−0.541
SI_Ke_	0.638	1	0.919	−0.907	−0.865
SI_Ke AI_	0.572	0.919	1	−0.917	−0.822
SI_Navarrete-Lopez_	−0.596	−0.907	−0.917	1	0.925
SI_Solak_	−0.541	−0.865	−0.822	0.925	1

**Table 9:** Comparison of the methods of quantification of segregation studies.

Method	Type	Scale	Correlation with the Other Methods ^1^	Advantages	Disadvantages	Materials Characterization	Origin of the Data	Results
**SI_UVP_**	Experimental	-	Moderate	- Easy data collection. - Materials characterization is not necessary.	- Less precision. - Does not analyze the data of the central zone of the sample.	- Not necessary	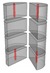	Segregation Index
**SI_Ke_**	Experimental	Is = 1.0: perfect uniformity. Is ≤ 0.95: start of segregation.	Very Strong	- The “vertical cut” of the specimens is not necessary.	- Does not analyze the data of the central zone of the sample. - Previous characterization of materials it´s necessary.	- Determination of the dry density of the upper and lower sections of the samples.	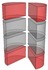	Segregation Index
**SI_KE IA_**	Image Analysis	Is = 1.0: perfect uniformity. Is ≤ 0.95: start of segregation.	Very Strong	- The “horizontal cut” of the specimens is not necessary.	- Does not analyze the data of the central zone of the sample. - Previous characterization of materials it´s necessary. - The accuracy of the method depends on the good distinction between the aggregates and the mortar matrix (image).	- Determination of the dry density of the mortar matrix. - Determination of the dry density of the LWAs.	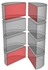	Segregation Index
**SI_Navarrete_**	Image Analysis	0%–40%—None to slight 40%–80%—Moderate 80%–120%—Severe 120%–160%—Slightly Stratified 160%–200%—Highly Stratified	Very Strong	- The “horizontal cut” of the specimens is not necessary. - Materials characterization is not necessary.	- Does not analyze the data of the central zone of the sample. - The accuracy of the method depends on the good distinction between the aggregates and the mortar matrix (image).	- Not necessary	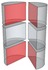	Segregation Index
**SI_IA_**	Image Analysis	0%—Homogeneous Distribution 100%—Maximum Segregation	Strong	- The “horizontal cut” of the specimens is not necessary. - Materials characterization is not necessary.	- The accuracy of the method depends on the good distinction between the aggregates and the mortar matrix (image).	- Not necessary	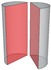	Segregation Index Segregation Profile ^2^

^1.^ Concept of the Evans Scale [52] applied to the average of the Pearson correlations among all other methods. ^2.^ Result presented in other publications of Solak [16] and Solak et al. [50].

**Figure A1:**
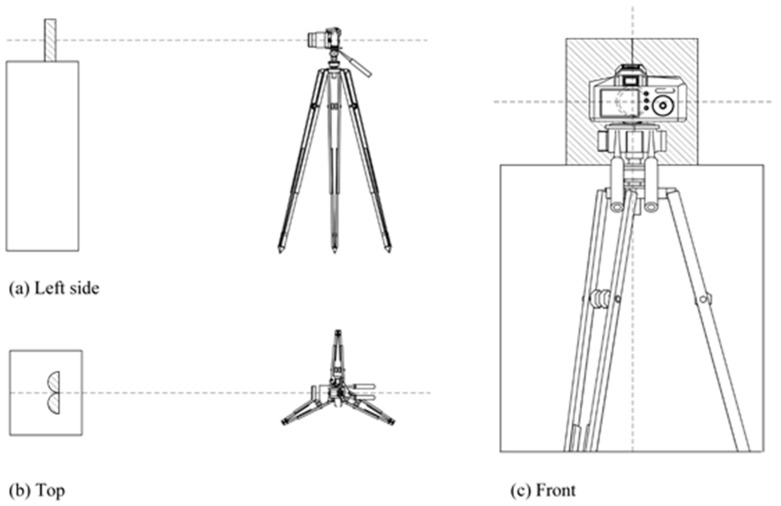
Alignment between samples and the camera lens axis. (**a**) Left side; (**b**) Top; (**c**) Front.

**Figure A2:**
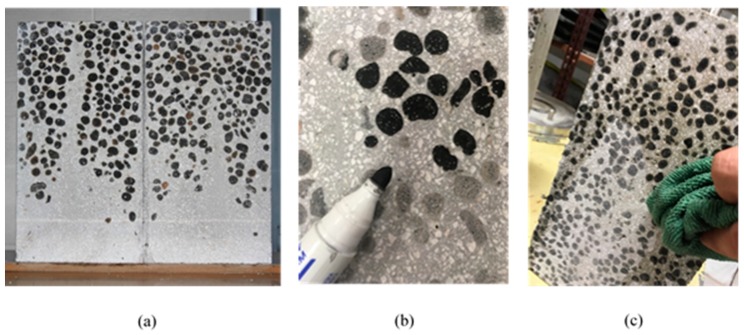
Recommendations for better data collection during photography. (**a**) halves of concrete specimen ready to be photographed; (**b**) permanent marker for highlighting the contrast; (**c**) moisten the surface to contrast the image.

## Supplementary Materials

The following are available online at https://www.mdpi.com/1996-1944/12/21/3642/s1: Figure S1: Minimum values of subsections required for a good accuracy (SI_IA_). Variation of the segregation index (SI_IA_) according to the number of sections for some samples used as an example. From 350 subsections (350 pixels in height), the values stabilize; Figure S2: Synthetic data: Rectangular samples, 200 elements, 10 (width) and 20 (height); Figure S3: Synthetic data: Squared samples, 100 elements, 10 (width) and 10 (height); Figure S4: Synthetic data: Rectangular samples, 200 elements, 20 (width) and 10 (height); Table S1: The results referring to the segregation indexes obtained for each concrete combination, vibration time, and manufacturing time are summarized in this table.

## Abbreviations

LWA	lightweight aggregate
LWAC	lightweight aggregate concretes
SI_Ke_	segregation index proposed by Ke [27,28]
SI_Ke IA_	segregation index proposed by Ke, obtained via image analysis
SI_Navarrete_	segregation index proposed by Navarrete et al. [33]
SI_IA_	segregation index proposed in this paper, using image analysis data
SI_UVP_	segregation index proposed by Solak et al. [39], using ultrasonic velocities data
GAI	Global Aggregate Index (determination of SI_IA_)
$N_{LWA}$	number of elements (pixels) classified as aggregates, found in a complete concrete cross-section (determination of SI_IA_)
$N_{M}$	number of elements (pixels) classified as mortar, found in a complete concrete cross-section (determination of SI_IA_)
LAI	Local Aggregate Index (determination of SI_IA_)
$n_{LWA_{i}}$	number of elements (pixels) classified as aggregates, found in a concrete subsection “i” (determination of SI_IA_)
$n_{M_{i}}$	number of elements (pixels) classified as mortar, found in a concrete subsection “i” (determination of SI_IA_)
LDC	Local Distribution Coefficient (determination of SI_IA_)

## Appendix A. Recommendations for Better Data Collection during Photography

### Appendix A.1. Alignment between Samples and the Camera Lens Axis

To ensure a greater accuracy of the method, we recommend that photographs be taken using a tripod or static support base. In this way, the lens axis coincides with the geometric center of the samples, both horizontally and vertically (Figure A1).

### Appendix A.2. Shoot both Halves at the Same Time

An interesting option for saving time and ensuring that the results of both halves of the same sample undergo similar treatments is to photograph both halves side by side at the same time (Figure A2a).

### Appendix A.3. Highlight one of the Material Fractions using Permanent Markers

When there is little or no color difference between the surface of the mortar matrix and the surface of lightweight aggregates, the use of permanent markers may highlight areas that correspond to a certain fraction of the material (Figure A2b).

### Appendix A.4. Moisten the Surface Before Shooting

To increase the contrast and allow a better distinction between the mortar matrix and light aggregates, the surface of the sample may be slightly moistened seconds before the photograph is taken (Figure A2c).

### Appendix A.5. Use White Cement

Where possible, replacing gray cement with white cement increases the contrast and allows a better distinction between mortar and aggregate phases.
