# Supplementary material for: Proposing a New Method Based on Image Analysis to Estimate the Segregation Index of Lightweight Aggregate Concretes
Source: Materials (Basel). 2019 Nov 5;12(21):3642. doi: 10.3390/ma12213642 (PMC6862299; doi:10.3390/ma12213642)
Supplement: Supplementary file 1 [file materials-12-03642-s001.pdf]

Supplementary Materials

Proposing a New Method Based on Image Analysis to Estimate the Segregation Index of Lightweight Aggregate Concretes

Afonso Miguel Solak [1,2], Antonio José Tenza-Abril [1], Francisco Baeza-Brotons [1] and David Benavente [3,*]

[1] Department of Civil Engineering, University of Alicante, 03080 Alicante, Spain; afonsosolak@gmail.com (A.M.S.); ajt.abril@ua.es (A.J.T-A.), fbaeza.brotons@ua.es (F.B-B)

[2] CYPE Ingenieros S.A., 03003 Alicante, Spain

[3] Department of Earth and Environmental Sciences, University of Alicante, 03080 Alicante, Spain

* Correspondence: david.benavente@ua.es

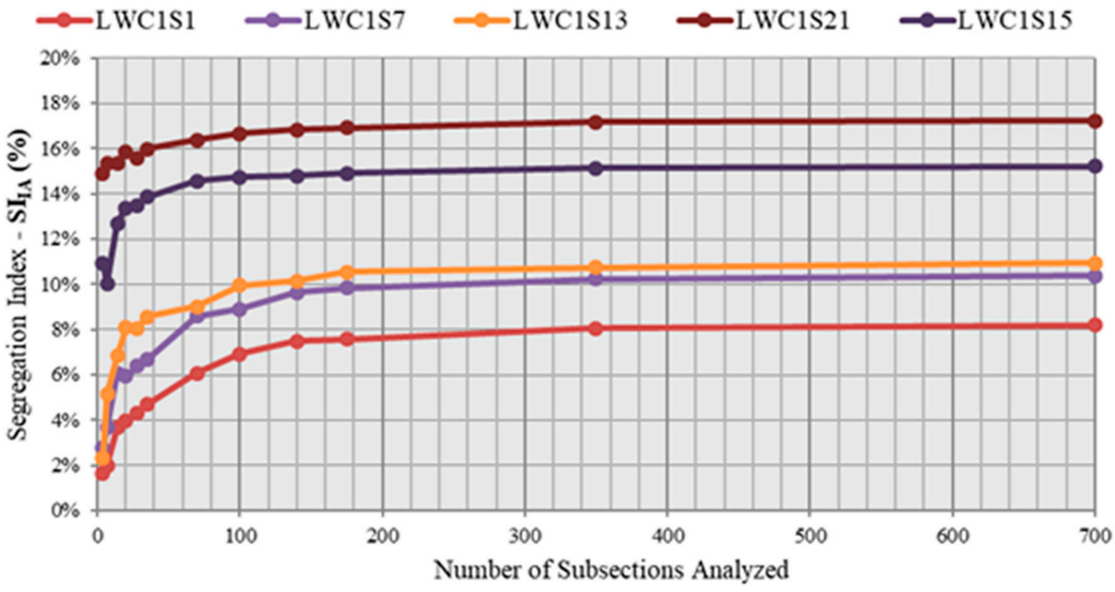

Figure S1. Minimum of subsections for good accuracy (SI_{IA}). Variation of the segregation index (SI_{IA}) according to the number of sections for some samples used as an example. From 350 subsections (350 pixels in height) the values stabilize.

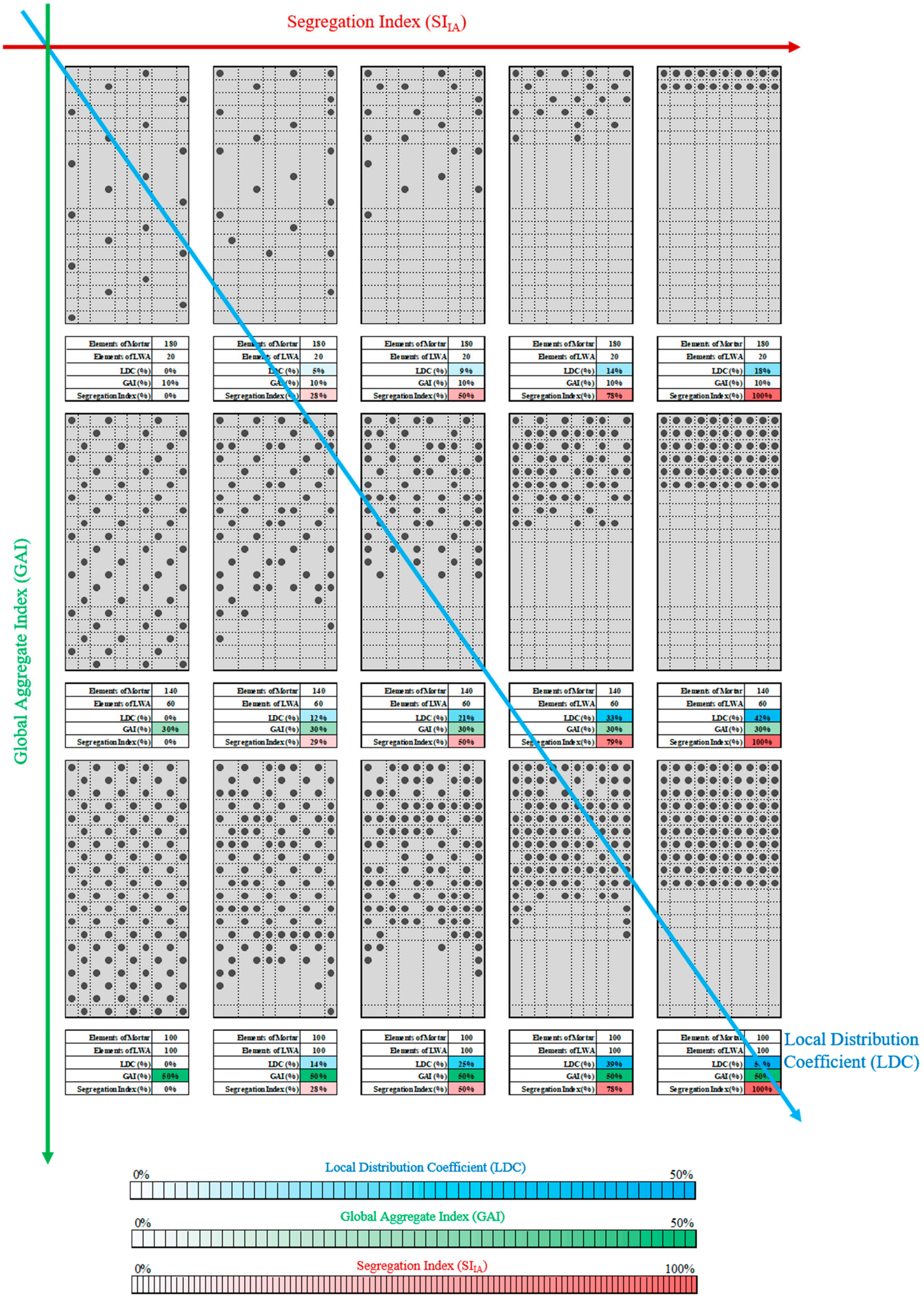

Figure S2. Synthetic data: Rectangular samples, 200 elements, 10 (width) and 20 (height).

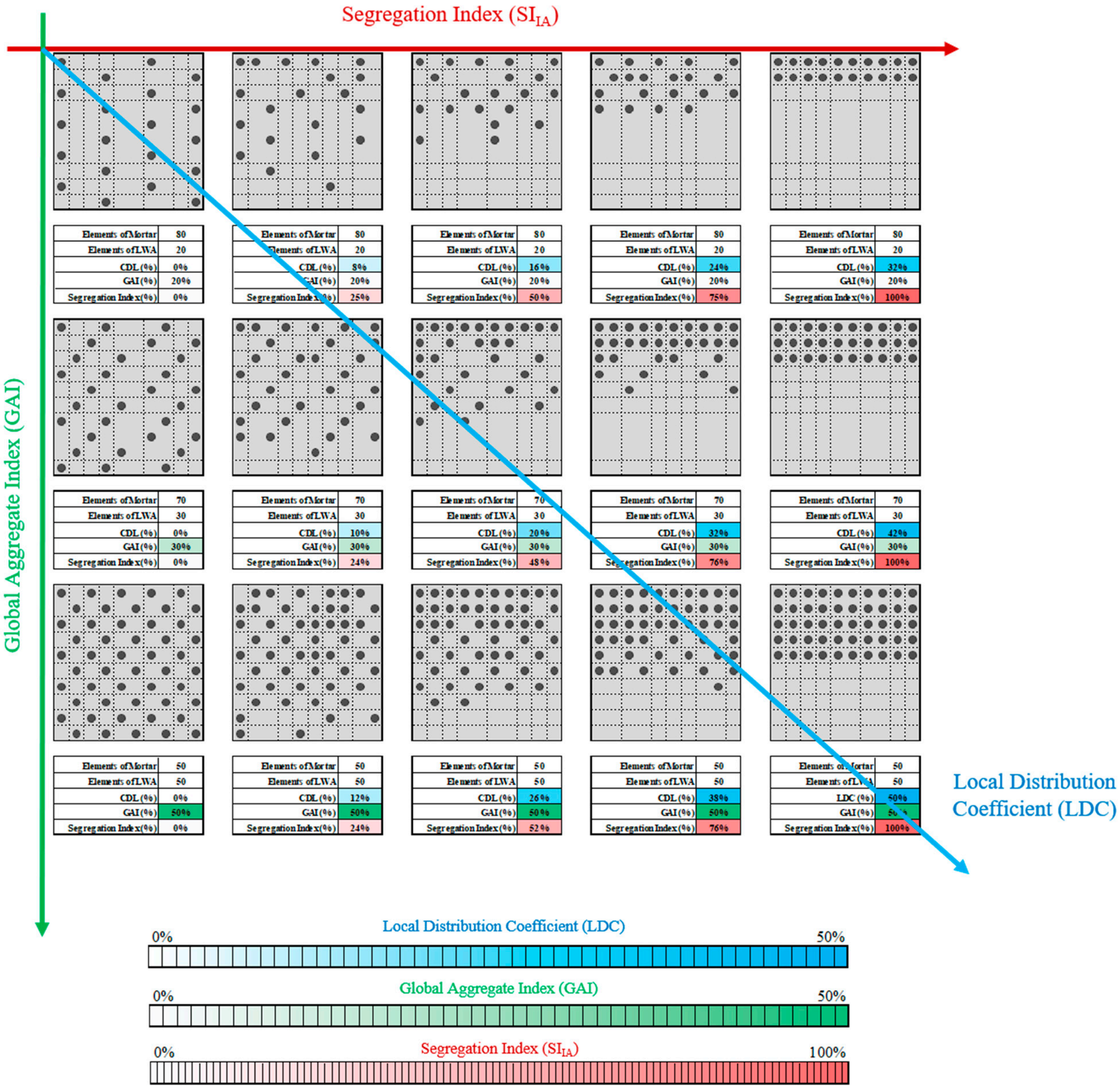

Figure S3. Synthetic data: Squared samples, 100 elements, 10 (width) and 10 (height).

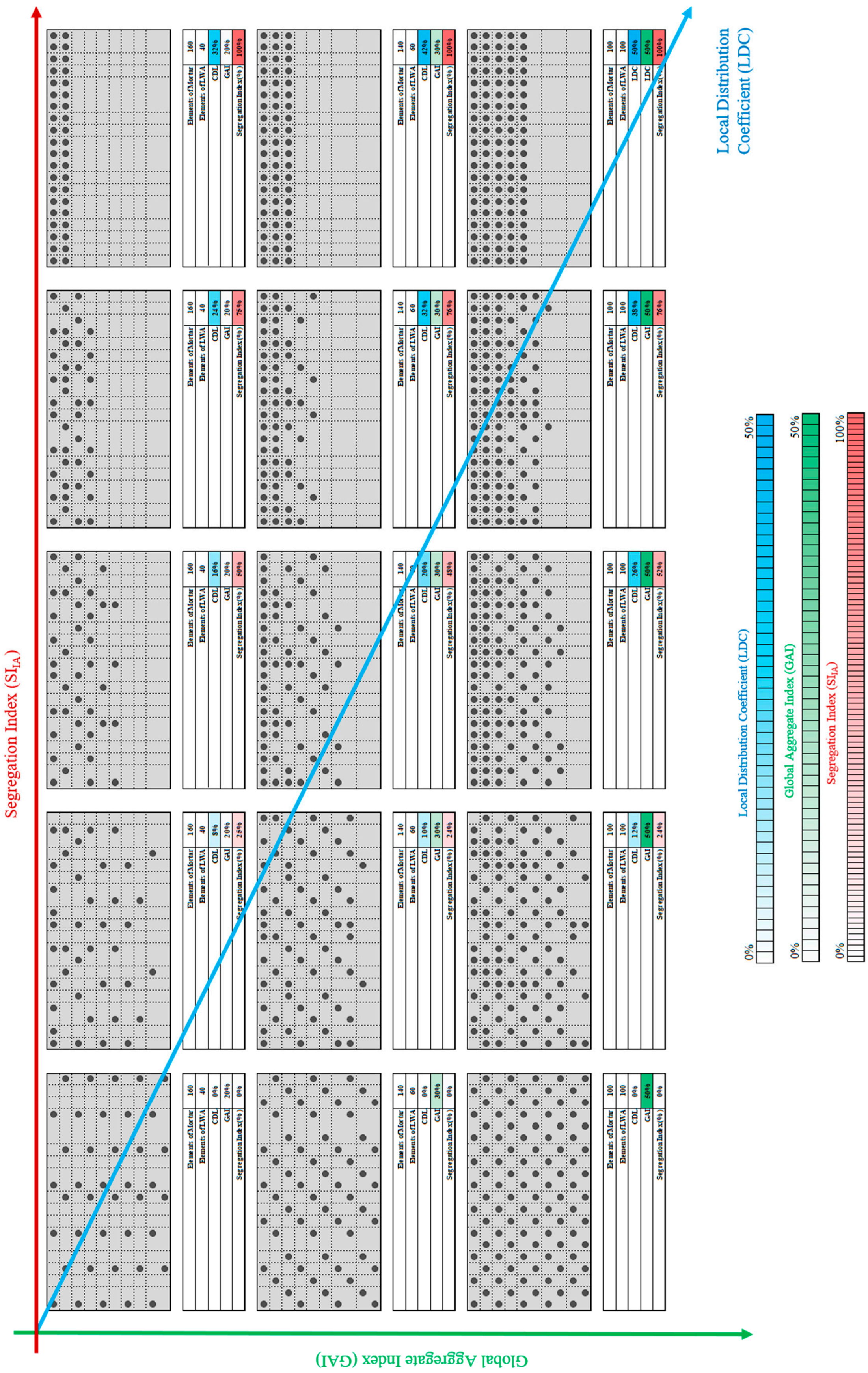

Figure S4. Synthetic data: Rectangular samples, 200 elements, 20 (width) and 10 (height).

Table S1. The results referring to the segregation indexes obtained for each concrete combination, vibration time and manufacturing time are summarized in this table.

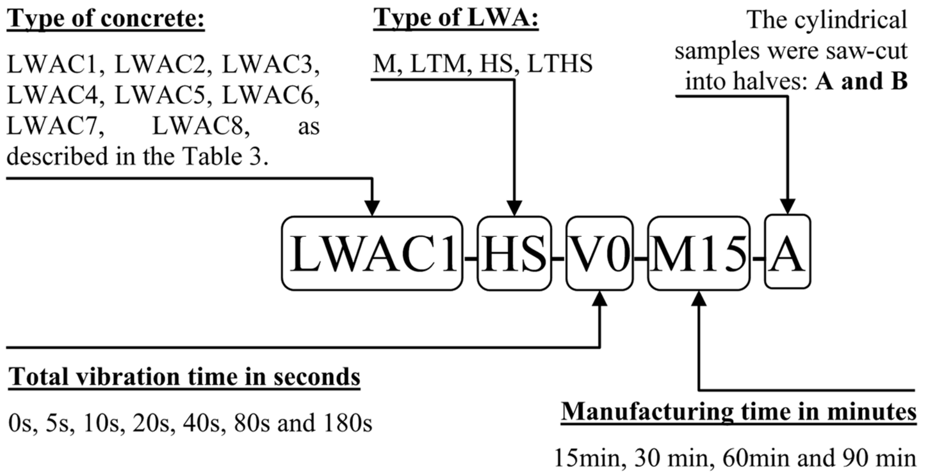

Sample Code	$SI_{Navarrete}$ (%)	SI_{IA} (%)	SI_{Ke}	$SI_{Ke\ IA}$	SI_{UPV}
LWAC1-HS-V0-M15-A	3.54	12.09	0.995	0.995	0.967
LWAC1-HS-V0-M15-B	4.19	14.03	0.995	1.019	0.974
LWAC1-HS-V0-M30-A	3.44	12.30	0.987	1.033	0.974
LWAC1-HS-V0-M30-B	40.41	21.49	0.979	0.825	0.967
LWAC1-HS-V0-M60-A	15.17	14.71	0.991	1.078	0.999
LWAC1-HS-V0-M60-B	17.49	14.02	0.969	1.072	0.957
LWAC1-HS-V0-M90-A	21.10	15.28	1.016	1.074	0.893
LWAC1-HS-V0-M90-B	30.15	14.57	0.991	1.115	0.950
LWAC1-HS-V10-M15-A	11.83	16.65	0.951	0.920	1.008
LWAC1-HS-V10-M15-B	1.77	17.42	0.960	0.961	1.017
LWAC1-HS-V10-M30-A	19.69	15.31	0.967	0.925	0.963
LWAC1-HS-V10-M30-B	20.30	14.20	0.948	0.916	0.963
LWAC1-HS-V10-M60-A	0.68	13.28	0.957	0.990	0.962
LWAC1-HS-V10-M60-B	11.67	14.91	0.962	0.958	1.033
LWAC1-HS-V10-M90-A	10.38	17.31	0.955	0.942	0.968
LWAC1-HS-V10-M90-B	7.76	14.05	0.942	0.962	1.014
LWAC1-HS-V20-M15-A	25.69	17.43	0.920	0.921	0.967
LWAC1-HS-V20-M15-B	13.18	15.09	0.913	0.968	0.898
LWAC1-HS-V20-M30-A	1.13	14.19	0.969	0.994	0.933
LWAC1-HS-V20-M30-B	11.22	13.68	0.971	1.038	0.978
LWAC1-HS-V20-M60-A	0.24	15.76	0.942	0.981	0.978
LWAC1-HS-V20-M60-B	0.01	16.83	0.917	0.984	0.949
LWAC1-HS-V20-M90-A	4.78	13.81	0.932	1.026	0.926
LWAC1-HS-V20-M90-B	2.10	13.94	0.951	0.987	1.062
LWAC1-HS-V40-M15-A	35.43	20.75	0.900	0.816	1.031
LWAC1-HS-V40-M15-B	26.63	17.91	0.892	0.869	0.966
LWAC1-HS-V40-M30-A	7.60	13.79	0.961	1.011	0.991
LWAC1-HS-V40-M30-B	10.89	16.34	0.919	0.940	0.939
LWAC1-HS-V40-M60-A	28.93	18.19	0.894	0.899	1.022
LWAC1-HS-V40-M60-B	43.70	18.47	0.914	0.856	0.917
LWAC1-HS-V40-M90-A	46.36	18.73	0.880	0.838	0.974

Sample Code	$SI_{Navarrete}$ (%)	SI_{IA} (%)	SI_{Ke}	$SI_{Ke\ IA}$	SI_{UPV}
LWAC1-HS-V40-M90-B	34.05	16.66	0.894	0.876	0.969
LWAC1-HS-V80-M15-A	49.98	22.81	0.841	0.792	0.977
LWAC1-HS-V80-M15-B	38.06	21.07	0.847	0.827	0.861
LWAC1-HS-V80-M30-A	28.89	19.68	0.912	0.870	0.968
LWAC1-HS-V80-M30-B	28.07	20.95	0.904	0.863	0.955
LWAC1-HS-V80-M60-A	26.63	17.91	0.850	0.869	0.954
LWAC1-HS-V80-M60-B	40.41	21.49	0.859	0.825	0.997
LWAC1-HS-V80-M90-A	58.31	21.69	0.847	0.832	0.880
LWAC1-HS-V80-M90-B	67.88	23.68	0.839	0.773	0.986
LWAC2-HS-V0-M15-A	5.40	16.11	1.002	1.001	0.999
LWAC2-HS-V0-M15-B	7.34	18.06	1.011	0.987	1.059
LWAC2-HS-V0-M30-A	2.94	16.73	0.988	0.994	1.009
LWAC2-HS-V0-M30-B	7.35	17.34	0.999	1.002	1.018
LWAC2-HS-V0-M60-A	4.24	18.89	1.016	1.010	1.046
LWAC2-HS-V0-M60-B	0.81	17.02	1.014	1.004	1.003
LWAC2-HS-V0-M90-A	9.91	15.71	0.998	0.985	1.051
LWAC2-HS-V0-M90-B	9.56	14.26	0.996	0.985	1.018
LWAC2-HS-V10-M15-A	2.16	19.91	0.985	1.015	0.988
LWAC2-HS-V10-M15-B	15.57	17.70	0.975	1.018	0.964
LWAC2-HS-V10-M30-A	8.25	15.84	0.988	0.990	0.977
LWAC2-HS-V10-M30-B	13.06	17.38	0.995	1.025	1.016
LWAC2-HS-V10-M60-A	10.57	17.57	0.977	1.018	0.961
LWAC2-HS-V10-M60-B	13.42	20.18	0.959	1.032	0.965
LWAC2-HS-V10-M90-A	38.33	19.22	0.995	1.071	0.973
LWAC2-HS-V10-M90-B	16.08	17.25	0.993	1.039	1.076
LWAC2-HS-V20-M15-A	1.92	16.68	0.952	0.980	1.000
LWAC2-HS-V20-M15-B	15.70	17.62	0.944	0.951	0.989
LWAC2-HS-V20-M30-A	2.39	18.92	0.940	0.992	0.991
LWAC2-HS-V20-M30-B	10.79	17.55	0.967	0.957	0.990
LWAC2-HS-V20-M60-A	25.84	16.82	0.925	0.939	0.912
LWAC2-HS-V20-M60-B	15.02	20.41	0.914	0.952	0.954
LWAC2-HS-V20-M90-A	12.12	19.31	0.980	1.000	0.988
LWAC2-HS-V20-M90-B	30.97	16.38	0.951	1.053	0.957
LWAC2-HS-V40-M15-A	29.57	18.89	0.937	0.931	0.963
LWAC2-HS-V40-M15-B	22.10	19.78	0.942	0.949	0.992
LWAC2-HS-V40-M30-A	34.65	23.99	0.944	0.922	0.987
LWAC2-HS-V40-M30-B	40.78	24.39	0.922	0.930	0.945
LWAC2-HS-V40-M60-A	37.49	17.81	0.922	0.899	0.935
LWAC2-HS-V40-M60-B	38.36	20.23	0.881	0.893	0.896
LWAC2-HS-V40-M90-A	17.27	22.65	0.959	1.002	1.008
LWAC2-HS-V40-M90-B	11.16	24.83	0.941	1.006	0.973
LWAC2-HS-V80-M15-A	56.47	21.42	0.905	0.890	0.955
LWAC2-HS-V80-M15-B	72.82	24.10	0.887	0.863	0.910
LWAC2-HS-V80-M30-A	60.00	26.62	0.915	0.859	0.927
LWAC2-HS-V80-M30-B	47.03	25.66	0.915	0.883	0.970
LWAC2-HS-V80-M60-A	50.72	21.54	0.873	0.863	0.891
LWAC2-HS-V80-M60-B	58.82	25.00	0.869	0.833	0.963
LWAC2-HS-V80-M90-A	11.04	26.88	0.942	0.952	0.952

Sample Code	$SI_{Navarrete}$ (%)	SI_{IA} (%)	SI_{Ke}	$SI_{Ke\ IA}$	SI_{UPV}
LWAC2-HS-V80-M90-B	18.54	28.62	0.925	0.933	0.984
LWAC3-M-V0-M15-A	15.09	15.23	1.036	1.038	0.924
LWAC3-M-V0-M15-B	5.43	18.53	0.951	0.973	1.030
LWAC3-M-V0-M30-A	3.60	16.08	0.978	0.993	0.981
LWAC3-M-V0-M30-B	3.60	16.08	0.984	0.993	1.045
LWAC3-M-V0-M60-A	0.56	15.81	0.993	1.013	0.959
LWAC3-M-V0-M60-B	24.95	19.10	1.008	0.942	1.030
LWAC3-M-V0-M90-A	9.48	16.18	0.944	0.939	0.931
LWAC3-M-V0-M90-B	13.51	15.36	1.025	0.971	0.976
LWAC3-M-V10-M15-A	19.38	18.32	0.916	0.907	1.000
LWAC3-M-V10-M15-B	18.90	18.86	0.895	0.898	0.940
LWAC3-M-V10-M30-A	23.37	21.84	0.708	0.885	0.971
LWAC3-M-V10-M30-B	24.89	18.64	0.922	0.892	0.951
LWAC3-M-V10-M60-A	22.79	16.98	0.870	0.910	0.984
LWAC3-M-V10-M60-B	42.45	22.58	0.934	0.850	1.011
LWAC3-M-V10-M90-A	10.94	17.65	0.971	0.966	0.981
LWAC3-M-V10-M90-B	5.26	17.80	0.990	0.999	0.952
LWAC3-M-V20-M15-A	43.64	19.99	0.751	0.850	0.936
LWAC3-M-V20-M15-B	69.87	22.12	0.926	0.813	1.013
LWAC3-M-V20-M30-A	52.95	21.94	0.891	0.838	0.839
LWAC3-M-V20-M30-B	48.83	26.44	0.888	0.818	0.969
LWAC3-M-V20-M60-A	55.03	19.79	0.876	0.828	0.895
LWAC3-M-V20-M60-B	55.80	21.79	0.917	0.836	1.025
LWAC3-M-V20-M90-A	34.26	22.16	0.903	0.854	0.936
LWAC3-M-V20-M90-B	19.59	18.92	0.896	0.925	1.010
LWAC3-M-V40-M15-A	123.77	36.79	0.744	0.624	0.979
LWAC3-M-V40-M15-B	116.86	34.37	0.877	0.694	0.880
LWAC3-M-V40-M30-A	133.89	36.20	0.703	0.655	0.907
LWAC3-M-V40-M30-B	126.26	34.24	0.730	0.714	0.992
LWAC3-M-V40-M60-A	110.74	31.42	0.806	0.687	0.944
LWAC3-M-V40-M60-B	107.26	30.76	0.808	0.701	0.907
LWAC3-M-V40-M90-A	79.52	25.98	0.843	0.750	0.940
LWAC3-M-V40-M90-B	86.80	27.81	0.909	0.757	0.946
LWAC3-M-V80-M15-A	160.27	42.01	0.595	0.575	0.820
LWAC3-M-V80-M15-B	173.42	45.09	0.644	0.537	0.921
LWAC3-M-V80-M30-A	161.50	42.63	0.621	0.597	0.874
LWAC3-M-V80-M30-B	170.13	43.95	0.602	0.550	0.858
LWAC3-M-V80-M60-A	142.67	37.71	0.590	0.603	0.873
LWAC3-M-V80-M60-B	137.44	37.34	0.661	0.608	0.879
LWAC3-M-V80-M90-A	142.08	39.74	0.685	0.596	0.923
LWAC3-M-V80-M90-B	138.58	38.86	0.691	0.601	0.928
LWAC4-M-V0-M15-A	11.79	21.91	1.010	1.011	1.017
LWAC4-M-V0-M15-B	40.25	23.68	0.994	1.044	1.021
LWAC4-M-V0-M30-A	21.26	20.53	1.004	1.043	1.047
LWAC4-M-V0-M30-B	40.78	25.66	0.996	1.050	0.937
LWAC4-M-V0-M60-A	3.89	21.41	0.975	0.991	1.082
LWAC4-M-V0-M60-B	9.62	19.40	0.966	0.992	1.015
LWAC4-M-V0-M90-A	15.63	21.65	0.982	0.950	1.059

Sample Code	$SI_{Navarrete}$ (%)	SI_{IA} (%)	SI_{Ke}	$SI_{Ke\ IA}$	SI_{UPV}
LWAC4-M-V0-M90-B	4.50	19.03	0.959	1.003	0.978
LWAC4-M-V10-M15-A	22.07	20.76	0.954	1.021	1.019
LWAC4-M-V10-M15-B	1.49	19.43	0.974	1.024	0.991
LWAC4-M-V10-M30-A	32.68	22.92	0.963	0.943	0.975
LWAC4-M-V10-M30-B	0.07	21.17	0.936	1.013	0.989
LWAC4-M-V10-M60-A	24.50	27.33	0.973	1.080	0.959
LWAC4-M-V10-M60-B	14.19	23.40	0.962	1.029	0.962
LWAC4-M-V10-M90-A	17.66	20.95	0.978	1.002	0.927
LWAC4-M-V10-M90-B	1.69	18.95	0.982	0.975	1.001
LWAC4-M-V20-M15-A	21.83	24.31	0.925	0.955	0.933
LWAC4-M-V20-M15-B	20.29	24.09	0.935	0.965	1.049
LWAC4-M-V20-M30-A	57.13	24.30	0.906	0.916	0.994
LWAC4-M-V20-M30-B	23.27	23.44	0.920	0.943	0.960
LWAC4-M-V20-M60-A	11.57	21.52	0.982	0.974	1.087
LWAC4-M-V20-M60-B	0.19	22.26	0.935	1.006	0.982
LWAC4-M-V20-M90-A	9.96	21.25	0.984	1.022	0.926
LWAC4-M-V20-M90-B	0.83	21.06	0.969	0.999	1.072
LWAC4-M-V40-M15-A	102.38	29.78	0.827	0.796	1.026
LWAC4-M-V40-M15-B	24.50	27.33	0.833	1.080	0.952
LWAC4-M-V40-M30-A	72.69	26.35	0.832	0.811	0.972
LWAC4-M-V40-M30-B	91.68	30.54	0.782	0.782	0.937
LWAC4-M-V40-M60-A	84.33	27.73	0.831	0.805	0.951
LWAC4-M-V40-M60-B	101.14	29.31	0.853	0.808	0.926
LWAC4-M-V40-M90-A	38.08	22.66	0.926	0.895	0.960
LWAC4-M-V40-M90-B	28.98	23.26	0.885	0.929	0.885
LWAC4-M-V80-M15-A	166.94	48.69	0.725	0.693	0.963
LWAC4-M-V80-M15-B	153.27	47.04	0.784	0.713	0.958
LWAC4-M-V80-M30-A	170.41	43.92	0.741	0.741	0.959
LWAC4-M-V80-M30-B	158.39	41.47	0.747	0.721	0.903
LWAC4-M-V80-M60-A	107.26	30.07	0.767	0.792	0.880
LWAC4-M-V80-M60-B	123.19	32.40	0.796	0.729	0.938
LWAC4-M-V80-M90-A	134.06	37.76	0.847	0.711	0.955
LWAC4-M-V80-M90-B	113.05	33.11	0.811	0.751	0.900
LWAC5-M-V10-M15-A	24.51	23.09	0.936	0.965	0.939
LWAC5-M-V10-M15-B	51.47	23.56	0.903	0.853	1.050
LWAC5-M-V160-M15-A	199.15	65.30	0.553	0.534	0.848
LWAC5-M-V160-M15-B	196.45	63.13	0.552	0.543	0.881
LWAC5-M-V20-M15-A	85.02	26.30	0.791	0.783	0.887
LWAC5-M-V20-M15-B	90.66	28.12	0.776	0.794	0.922
LWAC5-M-V40-M15-A	142.03	37.24	0.606	0.625	0.882
LWAC5-M-V40-M15-B	138.96	36.94	0.652	0.636	0.912
LWAC5-M-V5-M15-A	22.33	19.21	0.955	0.908	0.968
LWAC5-M-V5-M15-B	17.41	18.94	0.958	0.914	1.014
LWAC5-M-V80-M15-A	193.03	61.20	0.571	0.567	0.864
LWAC5-M-V80-M15-B	194.91	60.44	0.578	0.613	0.839
LWAC6-LTM-V10-M15-A	13.40	16.92	0.998	0.999	1.024
LWAC6-LTM-V10-M15-B	28.44	16.44	0.933	0.933	0.983
LWAC6-LTM-V160-M15-A	188.49	61.92	0.572	0.525	0.857

**Sample Code**	**SI$_{Navarrete}$ (%)**	**SI$_{IA}$ (%)**	**SI$_{Ke}$**	**SI$_{Ke\ IA}$**	**SI$_{UPV}$**
LWAC6-LTM-V160-M15-B	193.03	63.92	0.580	0.522	0.903
LWAC6-LTM-V20-M15-A	35.88	21.32	0.898	0.846	0.953
LWAC6-LTM-V20-M15-B	33.60	15.87	0.859	0.897	0.931
LWAC6-LTM-V40-M15-A	61.08	20.41	0.766	0.754	0.986
LWAC6-LTM-V40-M15-B	54.02	21.09	0.826	0.777	0.923
LWAC6-LTM-V5-M15-A	16.08	19.57	1.014	1.061	0.982
LWAC6-LTM-V5-M15-B	7.47	16.27	1.008	1.034	0.988
LWAC6-LTM-V80-M15-A	127.69	36.32	0.684	0.623	0.871
LWAC6-LTM-V80-M15-B	126.72	37.51	0.678	0.591	0.907
LWAC7-HS-V10-M15-A	6.80	15.26	1.033	1.000	1.013
LWAC7-HS-V10-M15-B	18.54	14.17	1.020	1.068	1.106
LWAC7-HS-V160-M15-A	56.53	22.38	0.820	0.779	1.003
LWAC7-HS-V160-M15-B	54.68	19.63	0.816	0.796	0.995
LWAC7-HS-V20-M15-A	3.64	10.90	1.036	0.968	1.073
LWAC7-HS-V20-M15-B	6.70	12.05	1.015	0.973	1.029
LWAC7-HS-V40-M15-A	17.55	12.45	0.983	0.921	0.989
LWAC7-HS-V40-M15-B	19.89	13.61	0.962	0.920	0.948
LWAC7-HS-V5-M15-A	0.05	12.63	1.023	1.008	1.014
LWAC7-HS-V5-M15-B	10.09	12.90	1.038	0.967	1.042
LWAC7-HS-V80-M15-A	41.83	18.96	0.920	0.831	0.908
LWAC7-HS-V80-M15-B	40.60	19.53	0.872	0.822	0.915
LWAC8-LTSH-V10-M15-A	10.54	11.68	1.048	1.047	0.989
LWAC8-LTSH-V10-M15-B	5.00	11.83	1.022	1.013	1.025
LWAC8-LTSH-V160-M15-A	88.32	30.59	0.759	0.693	1.034
LWAC8-LTSH-V160-M15-B	72.77	28.29	0.802	0.697	0.908
LWAC8-LTSH-V20-M15-A	0.33	10.97	1.008	1.003	1.098
LWAC8-LTSH-V20-M15-B	0.76	13.44	0.988	0.968	1.008
LWAC8-LTSH-V40-M15-A	47.37	19.11	0.943	0.852	0.966
LWAC8-LTSH-V40-M15-B	60.51	20.76	0.906	0.808	0.982
LWAC8-LTSH-V5-M15-A	3.69	13.69	1.007	1.033	1.027
LWAC8-LTSH-V5-M15-B	15.65	14.56	1.063	1.085	1.003
LWAC8-LTSH-V80-M15-A	48.45	19.66	0.840	0.790	0.978
LWAC8-LTSH-V80-M15-B	53.24	20.20	0.915	0.805	0.992
